# The Effect of Self-Care Training on Blood Sugar Control, HbA1C Level, and Life Quality of Diabetic Patients in Birjand, East of Iran: A Randomized Clinical Trial Study

**DOI:** 10.1155/2021/8846798

**Published:** 2021-01-21

**Authors:** Forough Ahrari, Zabihullah Mohaqiq, Mitra Moodi, Bita Bijari

**Affiliations:** ^1^School of Medicine, Birjand University of Medical Sciences, Birjand, Iran; ^2^Birjand University of Medical Sciences, Birjand, Iran; ^3^Department of Health Education, Faculty of Health, Birjand University of Medical Sciences, Birjand, Iran; ^4^Cardiovascular Diseases Research Center of Birjand University Medical Science, Birjand University of Medical Sciences, Birjand, Iran

## Abstract

**Background:**

As one of the most important public health problems worldwide, diabetes is closely linked with patients' lifestyles. The optimal approach to treating diabetes is to prevent it. Our aim in this study was to assess the impact of self-care behaviors on quality of life, blood sugar control, and HbA1C level in patients with type 2 diabetes.

**Methods:**

This randomized clinical trial examined 100 diabetic women referred to Ghadir Comprehensive Health Center in Birjand in 2019. A 5 cc fasting blood sample was taken from each participant. The participants were randomly assigned to experimental and control groups. For the experimental group, a 10-session self-care training workshop was held. Baseline and postintervention fasting blood glucose, HbA1C level, and life quality of the two groups were assessed and compared six months after the intervention. Data were analyzed in SPSS (16).

**Results:**

In the experimental group, the mean serum HbA1C level decreased from the baseline 7.5 ± 1.5 to 6.3 ± 1.0 (*P* < 0.001). Fasting blood sugar in the intervention group decreased from 136.3 ± 43.5 to 127.3 ± 22.9, but the reduction was not significant (*P*=0.322). The mean scores of the quality of life (*P*=0.002) and the visual analogue scale (*P* < 0.001*P* < 0.001) in the experimental group increased significantly compared to the control group.

**Conclusion:**

Self-care training for diabetic women had positive effects on both life quality and disease control. Therefore, it is recommended that self-care training be delivered and taken more seriously by physicians and health care providers in addition to drug therapy.

## 1. Introduction

Over time, changes in the lifestyle and diet of humans and the development of urbanization have led to an increased prevalence of chronic diseases. The burden of chronic diseases in developing countries is a serious threat. In the meantime, a highly important chronic disease that has affected public health worldwide is diabetes [1].

Diabetes is commonly associated with high blood sugar and insulin resistance or its decreased secretion and is the third leading cause of death across the world after cardiovascular disease and cancer [2, 3]. The prevalence of diabetes is increasing in developing and developed countries. Some studies have predicted that the disease will spread globally to 300 million people by 2025 [4]. According to reports published by the World Health Organization, the prevalence in Iran will reach more than 5 million by 2025 [5].

Diabetes is linked with some microvascular and macrovascular complications, which are associated with life-threatening disorders and reduced quality of life in diabetic patients [6]. Patients with diabetes run a 20-fold higher chance of developing cardiovascular disease and nephropathy than healthy people [7]. Blood sugar control can delay many of the acute and chronic complications of diabetes, especially those of type 2 diabetes [8]. Proper control of blood sugar and HbA1C has an effective role in reducing the delayed complications of diabetes; on the other hand, reduced self-care is associated with an increase in the disease complications [9, 10].

The disease is closely linked with lifestyle, nutrition, and physical inactivity. Prevention is the best approach to treating the disease because it does not have a definitive cure. Measures such as screening and diagnosis have led to improved quality of life and reduced costs for patients. On the other hand, timely identification and proper care based on patient education and self-care can prevent acute and chronic complications of the disease or delay the onset of complications [11]. Some studies have shown that patient education is conducive to improving self-care, reducing the risk of complications, and enhancing the quality of life [12].

Diabetes requires self-care behaviors of patients during the disease [12]. Increasing knowledge and improving attitudes toward diabetes is helpful in diet and diabetes control, which is called self-care [13]. Self-control is one useful way to control diabetes and blood sugar. Educational, behavioral, psychosocial, and clinical strategies are employed as a means of promoting and supporting self-control in diabetic patients [14]. The purpose of these strategies is to provide appropriate and effective methods for improving chronic diseases, mainly based on lifestyle changes, besides drug therapy, and other conventional treatments [15].

A study conducted in the city of Birjand on the determination of self-care predictors in type 2 diabetic patients showed that the patients were at a moderate level of knowledge and that self-efficacy and social support had the greatest impact on self-care [16].

Patients need education to understand their health status, make decisions about health care, and change health behaviors. Due to the specific geographical and cultural characteristics of this region (South Khorasan, Iran), Self-care of patients in this particular area and the factors affecting it may vary with other patients. Therefore, considering what went above and the high importance of life quality for diabetic patients, as well as the control and prevention of complications caused by this disease by implementing coherent educational programs, this study aimed to evaluate the impact of self-care behaviors on the quality of life, blood sugar control, and HbA1C level in patients with type 2 diabetes.

## 2. Material and Methods

### 2.1. Study Population

The study population involved patients with diabetes who referred to Ghadir Comprehensive Health Center in Birjand during 2019. Inclusion criteria were patients with diabetes referred to the health center, adult patients (age > 30), women, who had a medical record and were able to attend regular visits, informed consent to participate in the study, and the absence of another chronic illness. Exclusion criteria comprised an absence for more than two sessions from the training sessions or noncooperation with the researcher.

### 2.2. Sample Size and Sampling Technique

The sample size was computed as *n* = 45 per group based on Ahmadi et al.'s study [17], where the mean scores of HbA1C in the experimental and control groups were 7.78 ± 1.48 and 8.82 ± 2.11, respectively, using the formula for comparing means in two independent groups with 95% confidence and 80% power. Given an attrition rate of 10 percent, 50 subjects were considered per study group.

There were about 700 medical records of diabetic patients in Ghadir Comprehensive Health Center who were taking care of regularly on a monthly basis. They are visited once a month by health care providers and once every three months by a doctor.

The 110 patients who met the inclusion criteria of the study were invited telephonically for participation in the study and asked to visit the center on a specific day and time. The patients who visited were randomly assigned either to the experimental group or the control group by drawing lots. Five patients from each group were excluded from the study due to a lack of cooperation with the researcher, and, finally, the data of 100 patients (50 people in each group) were analyzed.

### 2.3. Study Design

This randomized clinical trial examined 100 diabetic women. The experimental group went through ten training sessions, comprising of session 1: fundamentals of the disease: definition, diagnosis, and symptoms of diabetes; session 2: therapeutic methods; session 3: the diet of diabetic patients; session 4: blood sugar measurement; session 5: physical activity; session 6: drug therapy of diabetic patients; session 7: patient follow-up; session 8: prevention and complications of diabetes; session 9: foot care; and session 10: questions and answers and a summary on diabetes. The training was delivered via lectures, using PowerPoint slides, educational videos, and questions and answers. At the end of each session, the contents were given to the patients as an educational pamphlet. The control group did not receive this education. After six months, the participants were contacted to visit the health center.

### 2.4. Measure of Variables

To measure fasting blood sugar (FBS) and glycosylated hemoglobin levels, a laboratory expert took 5 cc of blood from diabetic patients at 8:00 a.m. on an empty stomach (after 8 to 10 hours of fasting). Of the blood volume, 2 cc was discharged into a CBC tube for HbA1C measurement and 3 cc into a clotted SST for FBS measurement. The tubes were transferred to the specialized clinic laboratory of Imam Reza Hospital in Birjand to do the measurements.

To assess the quality of life of diabetic patients, the EQ-5D-5L questionnaire was used, which includes two parts. The first section comprises five dimensions (mobility, self-care, pain/discomfort, anxiety/depression, and usual activities) and one item for each dimension. A research colleague ticked the items on the questionnaire for the participants by interviewing them. The second section involves a visual scale numbered from 0 to 100, which is used to assess the quality of life. The patients were asked to rate their health on the scale. The above parameters were evaluated twice at baseline and end of the study.

The EQ-5D-5L questionnaire has been used in many studies to determine the quality of life in chronic diseases, and its validation has been confirmed in studies of chronic diseases such as diabetes. A study conducted on the quality of life of diabetic patients in Birjand reported it as a valid tool for assessing the quality of life of diabetic patients [18].

The height and weight of the patients were measured using a stadiometer in Ghadir Comprehensive Health Center. A research colleague assessed and recorded in a checklist patients' blood pressure levels after they had relaxed on a chair for five minutes.

### 2.5. Statistical Analysis

Data were entered into SPSS (16) software. After determining the normal distribution, data were analyzed with independent sample *t*-test, ANOVA, and Chi-Square tests. Mann–Whitney and Wilcoxon tests were employed for variables with an abnormal distribution (including quality of life and VAS). Statistical significance was inferred at (*P* < 0.05; *α* = 0.05).

### 2.6. Ethical Considerations

The protocol of this study was approved by the ethics committee of Birjand University of Medical Sciences, with the following ethical code: Ir.bums.rec.1398.94. All participants in the study signed written consent forms for participation.

## 3. Results

In this study, 100 female diabetic patients referred to a health center (*n* = 50) were assigned into a control group (without self-care training) and an experimental group (receiving self-care training). Based on the results of statistical experiments, there was no significant difference between the mean age (*P*=0.304), level of education (*P*=0.111), and the BMI level (*P*=0.080) in patients of the two groups (Table 1).

According to the results presented in Table 2, the mean blood pressure before and after the intervention did not differ significantly in the study groups (SBP: *P*=0.986 and DBP: *P*=0.970). The mean serum FBS level in the control group increased significantly (*P* < 0.001) over time; however, no significant change could be found for the experimental group (*P*=0.322). Also, the mean serum HbA1C level decreased significantly in the experimental group (*P* < 0.001); it did not change significantly in the control group (*P*=0.066).

Based on the results of Table 3, the mean score of quality of life in the experimental group increased significantly over time (*P*=0.002), while that of the control group showed no significant change (*P*=0.062). Moreover, the mean VAS score increased significantly in the experimental group, whereas it decreased significantly in the control group (*P* < 0.001).

The frequency distribution of life quality dimensions did not differ between the two groups before the intervention. However, the frequency distribution of motility (*P* < 0.001), self-care (*P*=0.037), and pain (*P*=0.002) in the experimental and control groups were significantly different, while other dimensions were not significantly different (*P* > 0.05) (Table 4).

## 4. Discussions

Today, due to the increasing burden of noncommunicable diseases in developing countries, especially diabetes, it has become ever more crucial to prevent such diseases and control them to prevent associated complications. On the other hand, given the highly important role of lifestyle in the development and progression of diabetes in patients, the importance of lifestyle modification in these patients has been increasingly highlighted [11]. Therefore, this study aimed to assess the effect of self-care training on blood sugar control, HbA1C level, and quality of life of diabetic patients.

The study showed that the mean FBS decreased in the experimental group over time, although the reduction was not statistically significant. In the studies by Zheng et al. [3] and Adu et al. [14], however, FBS in the experimental group decreased significantly at the end of the study, compared to the control group, which is not consistent with the findings of our study. Because of the fact that FBS depends on the duration of fasting, the quality and quantity of medication, and diets of patients, resulting in differences between sugar levels in individuals, this cannot be taken as highly important for the purposes of this study.

Based on the results of our study, the mean serum HbA1C level decreased significantly at the end of the study in the experimental group, while there was no significant change in the control group. In the studies conducted by Mahmoudi [19], Gagliardino et al. [20], and Ahmadi et al. [17], similarly, the mean serum level of HbA1C in the experimental group decreased significantly, with no significant change in the control group. Consistent with our study, Azizi et al. [21] indicated that postintervention mean serum levels of HbA1C were significantly lower in the experimental group than the control group. Decreased serum levels of this parameter in the patients suggested a better and more effective control of diabetes after the intervention. However, the controls, who built upon their basic level of knowledge, continued with their routine lifestyle, resulting in an unchanged average serum level of HbA1C.

The mean score of patients' quality of life in the experimental group was significantly higher in the experimental than in the control group, whereas the mean score of the two groups was not significantly different at baseline. In studies conducted by Shams et al. [22], Taghdisi et al. [23], and Zendetalab et al. [24], the mean score of quality of life in the experimental group overtook significantly that of the control group, whereas the baseline difference between the two groups was not significant. This is consistent with our study's findings.

Saeid Pour et al. [25] and Sharifirad et al. [26] reported similar baseline mean scores of quality of life in experimental and control groups. After the intervention, however, the experimental group scored significantly higher than the control group, which agrees with our findings. In line with our study, Hojjatollah et al.'s study (2016) reported that the mean score of life quality of diabetic elderly individuals in the experimental group was significantly higher than that of the control group at the end of the study [27]. Also, the studies conducted by Ganjlo et al. [28] and Mohammadi et al. [15] found that the mean score of quality of life in experimental group patients was significantly higher than that of the control group after intervention. Given that lifestyle changes and the associated significant effects on quality of life are time-consuming processes, it is predicted that patients may report better mean quality of life scores if their follow-up period would be longer.

Based on the results of this study, the frequency distributions of mobility, self-care, and pain in the two groups were significantly different. Also, in the experimental group, the mean score of mobility, self-care, usual activities, and pain/discomfort significantly decreased compared to the control group, while the mean score of anxiety and depression did not differ significantly between the two groups. Shams et al. [22] and Saeid Pour et al. [25] stated that the mean score of physical performance, physical role, body pain, and general health was significantly higher in the experimental group than the control group, which is consistent with our findings. However, in these studies, the mean score of patients' mental health in the experimental group was significantly higher than that of the control group, which is not in line with our study. Among the reasons for this discrepancy is that Shams et al.'s study covered mental health (including anxiety, stress, and depression), while our study examined the anxiety and depression of patients. Moreover, the duration of follow-up and the different types of interventions performed for patients should be noted.

Diabetes can affect all aspects of a patient's life. Given the results of our study indicating that diabetic patients suffer from anxiety, stress, and depression, it is recommended that these patients be examined on a yearly basis for mental health and be introduced to psychologists if needed.

### 4.1. Limitation

We have selected the participants from one health center which did not include all diabetic patients in the city; therefore, the results cannot be representative for all the diabetic patients in the city, which is one of the limitations of the study. A possible limitation of the study is the short duration (6 months) and the long-term effect of self-care on diabetes control and quality of life was not investigated.

## 5. Conclusion

The self-care training workshop for diabetic women demonstrated positive effects on diabetes control, including a significant reduction in serum FBS and HbA1C levels, as well as a significant increase in the mean quality of life and VAS scores. Therefore, it is recommended that self-care training be delivered and taken more seriously by physicians and health care providers in addition to drug therapy.

## Figures and Tables

**Table 1:** Comparison of demographic characteristics in study participants.

Study group	Status	Control group	Experimental group	*P* value
Mean age (year)		54.44 ± 8.85	52.86 ± 6.2	0.306

Education level frequency (percent)	Illiterate or elementary	25 (50.0)	16 (32.0)	0.111
	Secondary school	4 (8.0)	12 (24.0)	
	High school and associate	15 (30.0)	16 (32.0)	
	Bachelor or higher	6 (12.0)	6 (12.0)	

BMI level frequency (percent)	Normal	10 (20.0)	4 (8.0)	0.080
	Overweight	27 (54.0)	24 (48.0)	
	Obese	13 (26.0)	22 (44.0)	

**Table 2:** Comparison of mean blood pressure, FBS, and HbA1C in patients before and after the intervention.

Study group		Control group	Experimental group	*P* value
		(Mean ± SD)	(Mean ± SD)	
Systolic blood pressure (mmHg)	Before intervention	124.0 ± 16.6	112.2 ± 16.1	0.001
	After intervention	126.0 ± 15.4	111.6 ± 14.7	<0.001
*P* value		0.422	0.986	

Diastolic blood pressure (mmHg)	Before intervention	73.1 ± 12.9	66.0 ± 13.2	0.012
	After intervention	77.4 ± 13.9	65.7 ± 12.9	<0.001
*P* value		0.970	0.054	

FBS (mg/dl)	Before intervention	13.7 ± 37.9	136.3 ± 43.5	0.637
	After intervention	153.5 ± 45.9	127.3 ± 22.9	0.005
*P* value		<0.001	0.322	

HbA1C	Before intervention	6.9 ± 1.4	7.5 ± 1.4	0.028
	After intervention	7.21 ± 0.3	6.3 ± 1.0	0.001
*P* value		0.066	<0.001	

**Table 3:** Comparison of the mean score of quality of life and self-assessment of health before and after the intervention in the studied patients.

Study group		Control group	Experimental group	*P* value
		Median (Q%25–Q75%)	Median (Q%25–Q75%)	
Quality of life	Before intervention	0.80 (0.69–90)	0.77 (0.69–1)	0.900
	After intervention	0.75 (0.62–0.87)	0.89 (0.80–1)	<0.001
*P* value		0.062	0.002	

VAS (%)	Before intervention	80 (70–80)	80 (70–90)	0.465
	After intervention	70 (60–80)	85 (80–90)	<0.001
*P* value		<0.001	<0.001	

**Table 4:** Comparison of the frequency distribution of life dimensions after intervention in the studied patients.

Study group dimensions of the scale		Experimental number (percent)	Control number (percent)	*P* value
Mobility	No problems	37 (74%)	14 (28%)	<0.001
	Slight problems	112 (24%)	25 (50%)	
	Moderate problems	1 (2%)	4 (8%)	
	Severe problems	0 (0%)	6 (12%)	
	Unable to walk about	0 (0%)	1 (2%)	

Self-care	No problems	47 (94%)	38 (76%)	0.037
	Slight problems	3 (6%)	9 (18%)	
	Moderate problems	0 (0%)	2 (4%)	
	Severe problems	0 (0%)	1 (2%)	
	Unable to wash or dress	0 (0%)	0 (0%)	

Usual activities	No problems	42 (84%)	34 (68%)	0.193
	Slight problems	6 (12%)	12 (24%)	
	Moderate problems	2 (4%)	2 (4%)	
	Severe problems	0 (0%)	2 (4%)	
	Unable to do usual activities	0 (0%)	0 (0%)	

Pain/discomfort	No pain/discomfort	36 (72%)	22 (44%)	0.002
	Slight pain/discomfort	13 (26%)	14 (28%)	
	Moderate pain/discomfort	1 (2%)	9 (18%)	
	Severe pain/discomfort	0 (0%)	4 (8%)	
	Extreme pain/discomfort	0 (0%)	1 (2)	

Anxiety/depression	No anxiety/depression	36 (72%)	27 (54%)	0.231
	Slight anxiety/depression	9 (18%)	14 (28%)	
	Moderate anxiety/depression	5 (10%)	6 (12%)	
	Severe anxiety/depression	0 (0%)	2 (4%)	
	Extreme anxiety/depression	0 (0%)	1 (2)	

## Data Availability

The data used to support the findings of the study are provided within the article. All the data and materials are available upon reasonable request from the corresponding author.
